# A Mutation in the Mouse *Ttc26* Gene Leads to Impaired Hedgehog Signaling

**DOI:** 10.1371/journal.pgen.1004689

**Published:** 2014-10-23

**Authors:** Ruth E. Swiderski, Yoko Nakano, Robert F. Mullins, Seongjin Seo, Botond Bánfi

**Affiliations:** 1 Department of Anatomy and Cell Biology, Carver College of Medicine, University of Iowa, Iowa City, Iowa, United States of America; 2 Inflammation Program, Carver College of Medicine, University of Iowa, Iowa City, Iowa, United States of America; 3 Department of Ophthalmology and Visual Sciences, Carver College of Medicine, University of Iowa, Iowa City, Iowa, United States of America; 4 Department of Otolaryngology – Head and Neck Surgery, Carver College of Medicine, University of Iowa, Iowa City, Iowa, United States of America; 5 Department of Internal Medicine, Carver College of Medicine, University of Iowa, Iowa City, Iowa, United States of America; Washington University School of Medicine, United States of America

## Abstract

The phenotype of the spontaneous mutant mouse hop-sterile (hop) is characterized by a hopping gait, polydactyly, hydrocephalus, and male sterility. Previous analyses of the hop mouse revealed a deficiency of inner dynein arms in motile cilia and a lack of sperm flagella, potentially accounting for the hydrocephalus and male sterility. The etiology of the other phenotypes and the location of the *hop* mutation remained unexplored. Here we show that the *hop* mutation is located in the *Ttc26* gene and impairs Hedgehog (Hh) signaling. Expression analysis showed that this mutation led to dramatically reduced levels of the Ttc26 protein, and protein-protein interaction assays demonstrated that wild-type Ttc26 binds directly to the Ift46 subunit of Intraflagellar Transport (IFT) complex B. Although IFT is required for ciliogenesis, the Ttc26 defect did not result in a decrease in the number or length of primary cilia. Nevertheless, Hh signaling was reduced in the hop mouse, as revealed by impaired activation of Gli transcription factors in embryonic fibroblasts and abnormal patterning of the neural tube. Unlike the previously characterized mutations that affect IFT complex B, *hop* did not interfere with Hh-induced accumulation of Gli at the tip of the primary cilium, but rather with the subsequent dissociation of Gli from its negative regulator, Sufu. Our analysis of the hop mouse line provides novel insights into Hh signaling, demonstrating that Ttc26 is necessary for efficient coupling between the accumulation of Gli at the ciliary tip and its dissociation from Sufu.

## Author Summary

The Hedgehog (Hh) signaling pathway determines pattern formation in many developing tissues, e.g., during digit formation in the limbs, by regulating proteins of the Gli family. Activation of these proteins requires their transport to the tip of the primary cilium (an antenna-like sensory structure of the cell), and subsequent dissociation from their negative regulator, Sufu. Little is known about the mechanism underlying this dissociation. To gain new insights into Hh signaling, we analyzed the mutant mouse hop-sterile (hop), whose developmental defects suggest that the primary cilia are dysfunctional. We discovered that the *hop* mutation lies in the *Ttc26* gene, and that levels of the encoded protein are low in hop mice. Normal Ttc26 was found to bind to Intraflagellar Transport (IFT) complex B, a structure essential for building the cilium and moving proteins towards its tip. Nevertheless, unlike previously characterized mutations that affect IFT complex B, *hop* did not interfere with either the formation of primary cilia or the accumulation of Gli at their tips, but rather with dissociation of Gli from Sufu. Our results provide novel insights into Hh signaling, demonstrating that efficient coupling between Gli's accumulation at the ciliary tip and its dissociation from Sufu depends on Ttc26.

## Introduction

The Hedgehog (Hh) signaling pathway plays critical roles in embryonic development, wound healing, and tumorigenesis [1]–[4]. It is activated when the receptor protein Patched-1 (Ptch1) binds to one of the secreted Hh lipoproteins, Sonic Hh (Shh), Indian Hh, or Desert Hh [5], [6]. The Hh-Ptch1 interaction affects cell proliferation, differentiation, and patterning by regulating three Gli transcription factors (Gli1-3) [7], [8], and the extent of Gli activation is dictated by the ratio of the activator and repressor forms of Gli proteins in the cell. When Hh is absent, the full-length Gli3 protein (Gli3-F) is processed into a shorter repressor form (Gli3-R), which strongly suppresses the expression of target genes [9]. The repressor form of Gli2 (Gli2-R), in contrast, has only a minimal effect on transcription, and is formed inefficiently from full-length Gli2 (Gli2-F) [10]. Gli1 has no repressor form; it is regulated transcriptionally through activation of the other two Gli proteins [11]. Hh signaling activates both Gli2-F and Gli3-F and blocks their processing into repressors [9], [10]. Although data on the full range and importance of various posttranscriptional modifications of Gli are still emerging [12]–[16], it is clear that a crucial step in the activation of Gli2-F and Gli3-F is their dissociation from Sufu [17], [18].

The primary cilium is the Hh signaling center of the mammalian cell. In the absence of Hh proteins, Patch1 is localized to this structure, where it inhibits the activity of the seven-span transmembrane protein Smoothened (Smo) [19]. Activation of the Hh pathway causes Ptch1 to exit the cilium and Smo to enter [19], [20]. Smo enhances the ciliary import of Gli-F proteins (Gli-Fs) by inhibiting protein kinase A [21], [22], after which Gli-Fs are transported to the tip. This trafficking of Gli-Fs to the ciliary tip is required for their dissociation from Sufu [17], [18], and thus for activation of the Gli transcription factors [23]–[25].

Ciliary trafficking is facilitated by IFT particles, whose core proteins are organized into complexes A and B [26]. Complex B interacts physically with the kinesin-2 motor to mediate anterograde transport (towards the ciliary tip) [27], [28]. Complex A and cytoplasmic dynein 2 are necessary for retrograde transport. Mutations in the gene that encodes cytoplasmic dynein 2 lead to the production of shortened cilia and reduced Hh signaling [29], [30]. The complete absence of subunit Ift144 of IFT complex A likewise results in stumpy cilia and decreased Hh signaling, whereas a hypomorphic *Ift144* allele and the null alleles of two other genes encoding components of complex A (*Ift122*, *Ift139*) are associated with the formation of swollen cilia and enhanced Hh signaling [31]–[33]. With regard to mutations affecting complex B, depending on the subunit involved, defects can include a lack of ciliogenesis, the formation of short cilia, or the dysregulation of Ptch1 and Smo trafficking [24], [34]–[39]. All pathogenic mutations in complex B genes analyzed to date prevent or reduce Gli trafficking to the ciliary tip and inhibit Hh pathway activation. Two mechanisms by which complex B defects impair Hh signaling have been identified: indiscriminate interference with ciliary trafficking due to structural changes in the axoneme [40], or selectively dysregulation of re-localization of the Hh signaling proteins Ptch1 and Smo [34].

In an effort to gain additional insight into Hh signaling, we searched publicly available mouse lines with unidentified gene mutations for phenotypic signs of reduced Hh signaling. We selected the hop mouse for further analysis based on its preaxial polydactyly, hopping gait, hydrocephalus, and male sterility [41]–[47]; preaxial polydactyly also occurs in *Gli3^+/−^* mice [48], and a hopping gait has been described in *Gli1^−/−^; Gli2^+/−^* mice [49]. Furthermore, although Hh signaling and primary cilia had not been examined in hop mice previously, their motile cilia had been reported to be abnormal. Firstly, their sperms lack flagella, explaining the male sterility phenotype [43]. Secondly, although their epithelia in the trachea, oviduct, and ependyma form motile cilia, approximately 40% of these lack outer dynein arms [43]. Because ciliary beating at the apical side of ependymal cells is critical for the normal flow of cerebrospinal fluid [50], the partial lack of outer dynein arms in ependymal cilia likely explains the hydrocephalus of hop mice.

The *hop* mutation (also known as *hydrocephalic-polydactyly*, *hpy* [51]) arose spontaneously in an unirradiated mouse colony of the Radiobiology Unit of the Medical Research Council at Harwell more than 40 years ago [42]. Nevertheless, the gene harboring this mutation remained unidentified prior to our study. We used positional cloning to localize the *hop* mutation to the *Ttc26* gene, which encodes a component [52] of IFT complex B. Based on the association of Ttc26 with IFT complex B, we expected to find that the *Ttc26* mutation leads to the formation of abnormally short primary cilia, and to below-normal levels of Hh-triggered Gli accumulation at the ciliary tip. Surprisingly, neither proved true. Instead, Ttc26 was required for effective dissociation of Gli from Sufu. This finding suggests that Ttc26 protein is necessary for at least one step in the Hh pathway that lies downstream of Gli trafficking to the ciliary tip.

## Results

### The hop mouse harbors a nonsense mutation in the *Ttc26* gene

**Figure 1:**
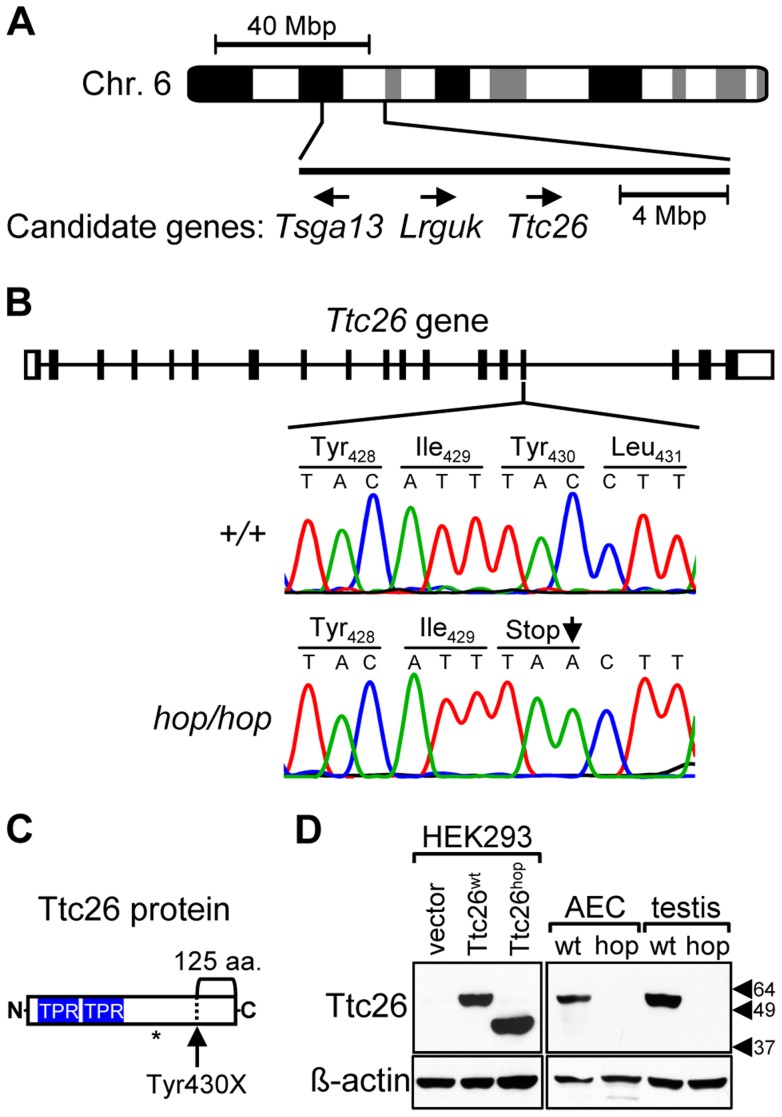
The *Ttc26* gene of the hop mouse contains a nonsense mutation. (**A**) Schematic representation of genomic positions of genes that both fall within the 16-mega base pair (Mbp) interval to which the *hop* mutation was mapped and had a known association with the ciliome. (**B**) Comparison of the 15^th^ exon of the *Ttc26* gene in wild-type and *hop/hop* mice. Horizontal lines represent introns, and black and white rectangles represent the coding and non-coding regions of exons, respectively. A deoxycytidine nucleotide (C) of wild-type *Ttc26* (upper chromatogram) is replaced with a deoxyadenosine (A) in the hop mouse, as indicated by an arrow in the lower chromatogram. The point mutation changes the tyrosine (Tyr) at position 430 of Ttc26 to a stop codon (Stop), as shown in the amino-acid sequence lines. (**C**) Schematic representation of the Ttc26 protein. Blue boxes indicate the predicted TPR motifs. The bracket indicates the C-terminal 125-amino acid region of the protein that is predicted to be missing in *hop/hop* cells. The asterisk indicates the position of the epitope that is recognized by the anti-Ttc26 antibody. (**D**) Immunoblot analysis of Ttc26 expression in transfected HEK293 cells, airway epithelial cells (AEC) and testis of wild type (wt) and *hop/hop* (hop) mice. HEK293 cells were transfected with the indicated Ttc26-encoding construct or an empty expression vector. Arrowheads indicate the positions of the 64, 49, and 37 kDa standards. The antibodies used for immunoblotting are indicated next to the upper and lower panels.

Although the gene affected by the *hop* mutation was not identified in previous studies, it had been localized to mouse chromosome 6 [47]. We hypothesized that the breeding history of the hop mouse line could facilitate further genetic mapping because the *hop* mutation had been transferred from an undefined genetic background onto the BALB/c background at The Jackson Laboratory, through repeated backcrosses. We analyzed chromosome 6 of hop mice for 15 SNPs that are almost completely BALB/c specific, as they have been detected in very few other strains (see Table S1). This approach identified a non-BALB/c region on chromosome 6 between SNPs rs36592952 and rs30852324 (Figure 1A), encompassing 142 protein-coding RefSeq genes. We selected 3 of these genes for testing (Figure 1A), based on their known association with the ciliome [53], [54]. Sequence analysis of the candidate genes from the hop line showed that one, *Ttc26*, contained a C-to-A point mutation that changes a tyrosine-encoding codon to a stop codon in exon 15 (Figure 1B). This nonsense mutation is predicted to truncate the Ttc26 protein 125 amino acids from its C-terminus (Figure 1C). We used Western blotting to examine expression of the wild-type Ttc26 protein (Ttc26^wt^) as well as the hop genome-encoded Ttc26 (Ttc26^hop^). Each was readily detected in HEK293 cells transfected with the corresponding construct, and Ttc26^hop^ was ∼13 kDa smaller (Figure 1D). Immunoblotting also revealed the presence of endogenous Ttc26^wt^ in airway epithelial cells and the testes of wild-type mice, but Ttc26^hop^ was not detected in the equivalent samples from hop mice (Figure 1D). We hypothesized that the premature stop codon in the endogenous Ttc26^hop^ mRNA may trigger nonsense-mediated decay (NMD), but that the transfected and intronless Ttc26^hop^ evades degradation because NMD is not activated by a premature stop codon in the absence of downstream exon-exon junctions [55]. Real-time RT-PCR experiments revealed that, in mouse embryonic fibroblasts (MEFs) from the hop line, expression of the endogenous Ttc26^hop^ mRNA was reduced 5.7-fold compared to that of the Ttc26^wt^ mRNA in control MEFs (Figure S1). These results strongly suggest that the *hop* mutation is located within the *Ttc26* gene, and that it leads to reduced expression of the Ttc26 protein.

### The *hop* mutation is associated with partial embryonic lethality and patterning defects

**Figure 2:**
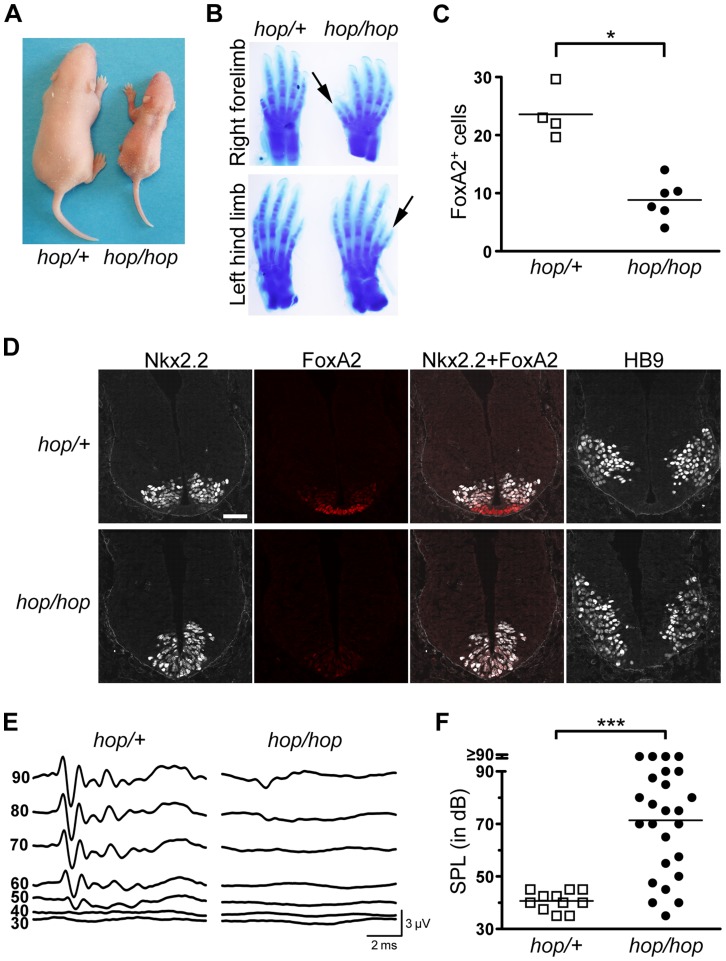
The hop mouse exhibits patterning defects and hearing impairment. (**A**) Representative images of *hop/+* and *hop/hop* mice at postnatal day 4. (**B**) Comparison of Alcian Blue-stained fore and hind limbs of *hop/+* and *hop/hop* mice. Extra digits are indicated by arrows. (**C**) Statistical analysis of FoxA2-positive cells in the lumbar neural tubes of *hop/+* and *hop/hop* mice (E10.5). Each symbol represents the average number of FoxA2^+^ cells per focal plane in a single embryo (for each embryo, 12 focal planes in 4 sections were analyzed, Mann-Whitney test: *P = 0.01). (**D**) Immunostaining of the lumbar neural tube of *hop/+* and *hop/hop* mice (E10.5) with antibodies against the V3 progenitor marker Nkx2.2 (white contrast), the floor plate marker FoxA2 (red), and the motor neuron protein HB9 (white contrast). The *hop/hop* genotype is associated with reduced FoxA2 expression and ventralization of the Nkx2.2- and HB9-expressing cells. Scale bar: 50 µm. (**E**) Representative ABR waveforms for 3–4 week-old *hop/+* and *hop/hop* mice. Broadband click stimuli were applied at the indicated sound pressure levels (in dB). (**F**) Statistical analysis of ABR thresholds measured in 3–4 week-old *hop/+* and *hop/hop* mice. Broadband click stimuli between 30 and 90 dB sound pressure level (SPL) were used. Each symbol represents the value for a single mouse (Mann-Whitney test: ***P<0.0001).

We found that each homozygous *Ttc26* mutant mouse (n = 82) in our hop colony had preaxial polydactyly (Figure 2B). Thus, the previously reported polydactyly phenotype [44], [47] was fully penetrant in this line. However, we observed that most litters included fewer *hop/hop* mice than expected based on Mendelian ratios. The genotyping of 126 newborn mice from heterozygous breeding pairs confirmed that the ratio of *hop/hop* mice was significantly lower than the expected 25% (33 wild-type [26.2%], 75 heterozygous [59.5%], and 18 homozygous mutant [14.3%]; *χ^2^* test p = 0.017). In contrast, when embryos were harvested from heterozygous breeding pairs on embryonic day (E) 10.5, the ratio of *hop/hop* mice was close to 25% (27 wild-type [26.2%], 51 heterozygous [49.5%], and 25 homozygous mutant [25.7%]; *χ^2^* test p = 0.96). Thus, homozygosity for the *Ttc26* mutation is associated with partial lethality between E10.5 and birth. The surviving homozygous *Ttc26* mutant mice were smaller than control littermates (Figure 2A), consistent with a previous characterization of the hop mouse line [44].

One of the best-studied examples of Shh-regulated patterning besides digit formation in the limb is cell-type specification in the neural tube. In this structure, neuronal fates are dictated by the local concentration of Shh, which decreases gradually from its source in the notochord and floor plate [28], [56], [57]. We examined the pattern of neuronal specification in the ventral neural tubes of *hop/hop* and control mice based on the expression of three markers of neuronal cell types: FoxA2, Nkx2.2, and HB9. Immunofluorescence detection of FoxA2 at E10.5 revealed that the number of FoxA2-positive cells (which require the highest Shh concentration for their specification) was reduced in the neural tubes of *hop/hop* mice compared to that in their heterozygous littermates (Figures 2C and 2D). Furthermore, in the *hop/hop* mice the Nkx2.2-expressing V3 interneurons were shifted to the ventromedial region (Figure 2D), and the ventral edge of the HB9-expressing motoneuron area was located abnormally close to the ventral border of the neural tube (Figure 2D). Thus, the *Ttc26* mutation is associated with patterning defects that are characteristic of reduced Hh signaling.

Defects in the primary cilium often lead to hearing loss, polycystic kidney disease, and retinopathy [58]. Testing of the auditory brainstem responses (ABR) of *hop/hop* and heterozygous control mice to broad-band sound stimuli of various intensities revealed that the homozygous animals were hearing impaired (Figure 2E and 2F); however, the severity of the hearing impairment was variable. Histological examination of the cochlear cross sections of hearing impaired *hop/hop* mice did not identify pathological changes other than reduced thickness of the bony labyrinth (Figure S2). Furthermore, visualization of the bundles of stereocilia in whole-mount preparations of organ of Corti samples from *hop/hop* mice showed that the planar orientation of hair cells was not altered (Figure S3). Thus, unlike the lack of several other ciliary proteins [59], [60], the Ttc26 defect of hop mice impairs hearing through mechanisms other than the dysregulation of planar polarity in hair cells. Histological examination of the kidneys and retinas of 1 year old *hop/hop* mice also did not reveal abnormalities (Figure S4). Collectively, these results indicate that the *Ttc26* mutation is associated with some – but not all – of the pathological changes that are typically linked to dysfunction of the primary cilium.

### Full-length Ttc26 interacts directly with the Ift46 subunit of IFT complex B

**Figure 3:**
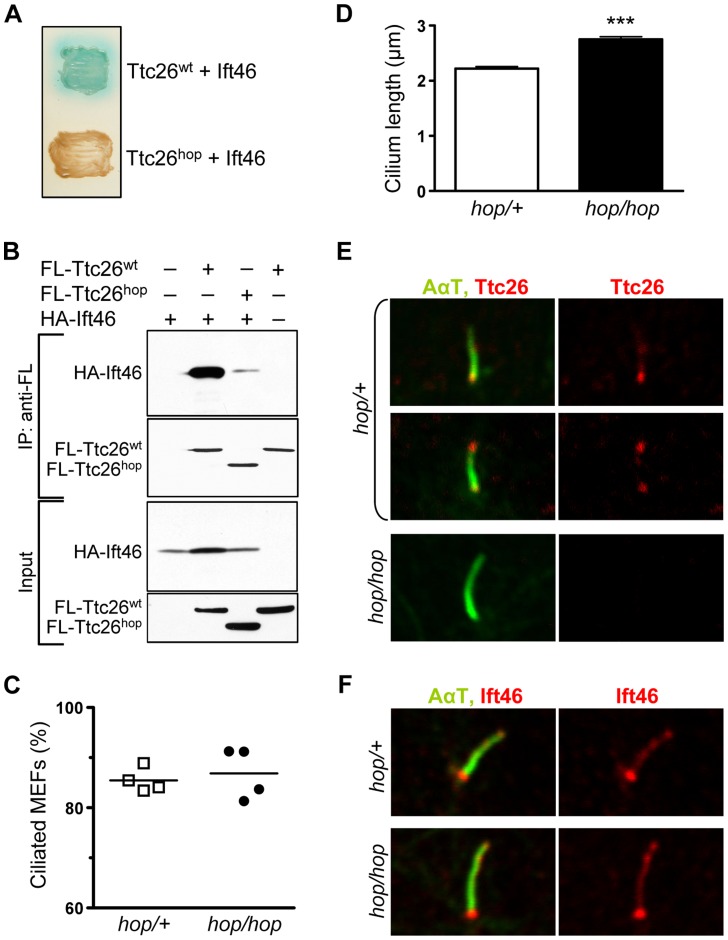
The *hop* mutation does not impair ciliogenesis or ciliary localization of the Ttc26-interacting protein Ift46. (**A**) Color test of protein-protein interactions in yeast transformed with the indicated combination of Ift46 and Ttc26^wt^ or Ttc26^hop^. Blue staining of the yeast colony (upper patch) is indicative of a protein-protein interaction; a lack thereof indicates the absence of a protein-protein interaction (lower patch). (**B**) Immunoprecipitation analysis of the Ttc26^wt^-Ift46 interaction. HEK293 cells were transfected with the indicated combinations of HA-tagged Ift46 and flag (FL)-tagged Ttc26^wt^ or Ttc26^hop^. The two upper panels show immunoblot analysis of tagged proteins pulled down with an anti-flag antibody, and the two lower panels show immunoblot analysis of input controls. (**C**) Percentages of ciliated *hop/+* and *hop/hop* MEFs following 2 days of serum starvation. MEFs were isolated from 4 embryos per genotype, and 150 MEFs per embryo were analyzed. (**D**) Statistical analysis of cilium length in the primary cultures of serum-starved *hop/+* and *hop/hop* MEFs (mean ± SEM, n = 200 cilia per genotype, unpaired *t*-test with Welch correction, ***P<0.0001). (**E,F**) Immunofluorescence analysis of (**E**) Ttc26 and (**F**) Ift46 expression in the cilia of *hop/+* and *hop/hop* MEFs. The axoneme was visualized by immunolabeling of acetylated-α-tubulin (AαT).

Although Ttc26 had been co-purified with IFT complex B and was recently classified as a subunit of this complex [52], [61], [62], its ability to interact directly with proteins had not been examined. To gain insight into Ttc26 function, we screened mouse and human cDNA libraries for Ttc26^wt^-binding proteins, using the yeast two-hybrid method. These screens identified a single Ttc26-interacting protein: the IFT complex B subunit Ift46. Next, we tested whether the C-terminus of Ttc26 is required for this protein-protein interaction, using the two-hybrid assay in yeast co-transformed with Ift46 and Ttc26^wt^ or Ift46 and Ttc26^hop^. Because Ift46 was fused to a Gal4 activator domain and the Ttc26 proteins were fused to a Gal4 DNA-binding domain, the Ift46-Ttc26 interaction was predicted to reconstitute the active Gal4 transcription factor. We visualized Gal4 activity by supplementing the yeast plates with X-α-Gal, which is metabolized into a blue product by a Gal4-induced enzyme. This test showed Gal4 activation in yeast co-transformed with Ift46 and Ttc26^wt^, but not in yeast co-transformed with Ift46 and Ttc26^hop^ (Figure 3A). Thus, the Ttc26 C-terminus is required for binding to Ift46.

We used immunoprecipitation to test whether Ift46 and Ttc26 can interact in mammalian cells. HEK293 cells were transfected with HA-tagged Ift46 and either flag-tagged Ttc26^wt^ or flag-tagged Ttc26^hop^. The cells were lysed, and Ttc26 and the interacting proteins were pulled down from the lysates using an anti-flag antibody. Immunoblot analyses of the proteins in these samples confirmed that Ift46 interacted efficiently with Ttc26^wt^ (Figure 3B) but not with Ttc26^hop^, supporting the idea that the Ttc26 C-terminus is important for this interaction (Figure 3B, see quantification in Figure S5). Thus, even if the endogenous Ttc26^hop^ protein is expressed in the hop mice at levels below the detection limit of our Western blots, its ability to interact with Ift46 is severely impaired.

### The *hop* mutation does not lead to the shortening of cilia in embryonic fibroblasts

Defects in most subunits of IFT complex B cause either a complete lack of ciliogenesis or the formation of short cilia, depending on the affected subunit [27]. We therefore evaluated cilium formation in the MEFs of *hop/hop* and wild-type mice, visualizing the ciliary marker proteins acetylated-α-tubulin (AαT) and Arl13b by immunofluorescence. We normalized the number of visualized cilia to the number of ToPro-3-stained nuclei. These experiments revealed that the *hop* mutation did not affect the percentage of ciliated MEFs (Figure 3C). Furthermore, it did not result in the production of short cilia, in either MEF cultures (Figure 3D) or the mesenchyme of *hop/hop* embryos (Figure S6). In fact, the *hop* mutation was associated with a slight increase in cilium length (Figure 3D and Figure S6). Next, we evaluated the expression of Ttc26 and Ift46 in the cilia of control and *hop/hop* MEFs by immunofluorescence. In control MEFs, Ttc26 was detected at the base of most cilia and at the tip of ∼20% of cilia (Figure 3E); the specificity of the immunostaining was confirmed by the lack of Ttc26 signal in the cilia of *hop/hop* MEFs. The expression of Ift46 was not different in the cilia of control and *hop/hop* cells (Figure 3F). Collectively, these results show that the *Ttc26* mutation of hop mice does not inhibit the formation of primary cilia or the ciliary localization of Ift46.

### Gli activation is reduced in the embryonic fibroblasts of hop mice

**Figure 4:**
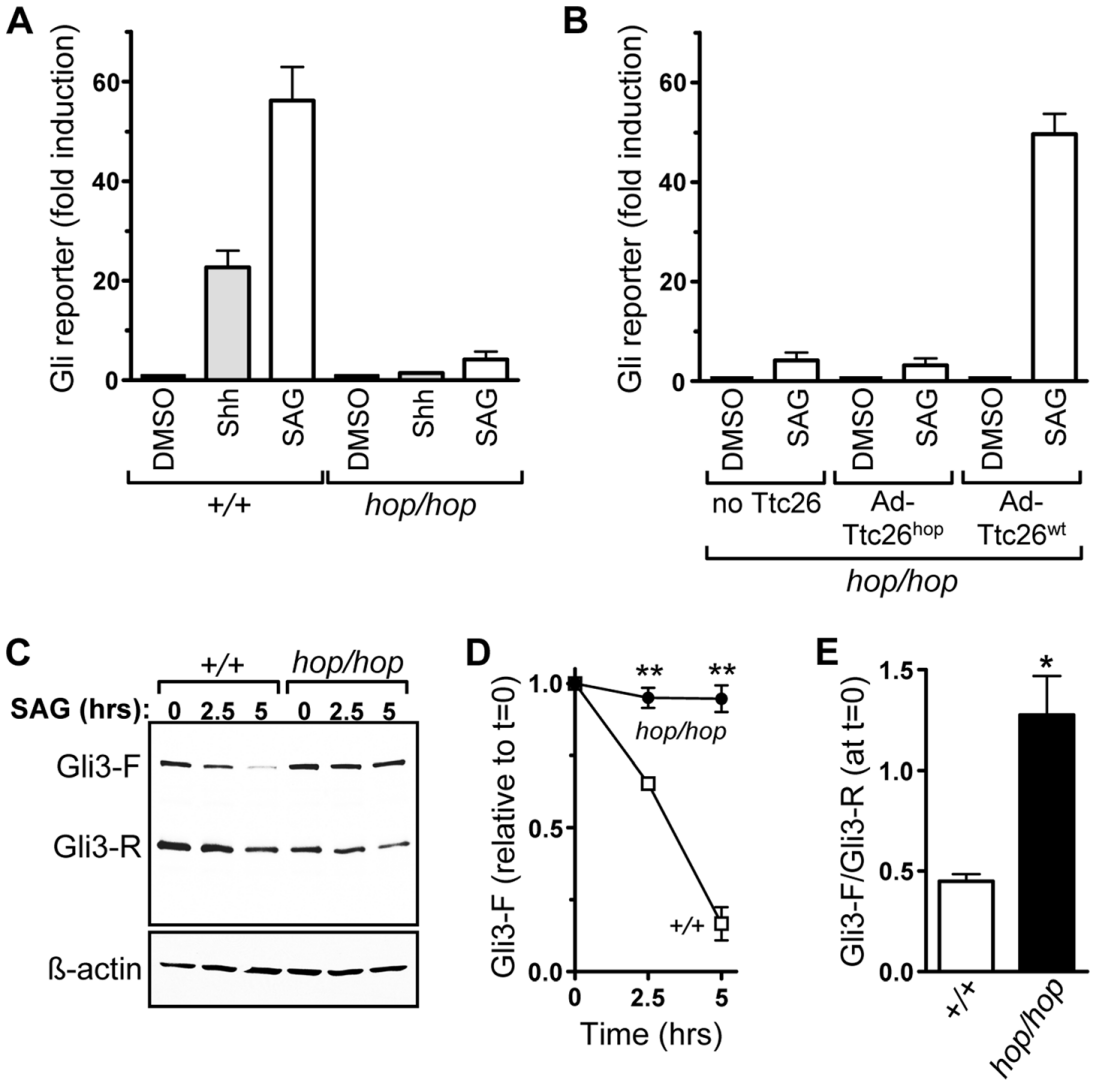
The *Ttc26* mutation in the hop mouse impairs Shh signaling. (**A**) Induction of an adenovirus-delivered Gli^x^8-luciferase reporter gene in wild-type (+/+) and *hop/hop* MEFs following 2-day incubation with DMSO (0.02%), Shh-conditioned medium, or SAG (400 nM) as indicated (mean ± SEM, n = 3–7). The increase in luciferase expression is shown relative to that in the DMSO control. Constitutive GFP expression from the Gli^x^8-luciferase-encoding viral vector was used for normalization. (**B**) SAG-dependent induction of the Gli^x^8-luciferase gene in *hop/hop* MEFs following adenoviral delivery of Ttc26^hop^ or Ttc26^wt^ (mean ± SEM, n = 3–7). The control group of MEFs was not transduced with a Ttc26-encoding virus (no Ttc26). The luciferase signal was normalized to GFP expression as described in panel A. (**C**) Immunoblot analyses of expression of Gli3-F and Gli3-R in wild-type and *hop/hop* MEFs, following SAG treatment (400 nM) for the indicated times. ß-actin serves as a loading control (lower panel). (**D**) Statistical analysis of Gli3-F band intensities in the immunoblot experiments described in panel C (mean ± SEM, n = 3; two-way ANOVA, *P*<0.006 for the genotype variable; *post-hoc* Bonferroni test, **P<0.01). (**E**) Statistical analysis of the ratio of the Gli3-F and Gli3-R bands at the 0 time point in the experiments described in panel C (mean ± SEM, n = 3; unpaired *t*-test, *P = 0.014).

The patterning defects in the limbs and neural tube of *hop/hop* mice suggested that the *Ttc26* mutation impairs Hh signaling. To evaluate the functionality of the Hh pathway, we transduced control and *hop/hop* MEFs with an adenovirus that encodes a previously described Gli-responsive reporter gene [63]–[65], and measured reporter expression following 48-h treatment with Shh-conditioned medium. Gli reporter signal was normalized to the expression of GFP from a separate expression cassette in the adenoviral vector. This experiment revealed that Shh-induced expression of the Gli reporter was dramatically reduced in the *hop/hop* MEFs compared to wild-type controls (Figure 4A). Next, we tested the response of control and *hop/hop* MEFs to the chemical agonist SAG, which activates Smo directly and induces Shh signaling independently of Ptch1 [66]. Again, induction of the Gli reporter was much lower in *hop/hop* MEFs than in their control counterparts (Figure 4A). Thus, the *hop* mutation impairs Hh signaling downstream of Ptch1.

We also used the Gli reporter assay to assess whether the *Ttc26* mutation was the cause of the Hh signaling defect in *hop/hop* cells. MEFs from *hop/hop* mice were co-transduced with the Gli reporter-encoding virus and either a Ttc26^wt^- or Ttc26^hop^-encoding adenovirus, and *hop/hop* MEFs transduced with only the Gli reporter-encoding virus served as a negative control. Western blot analysis confirmed that the Ttc26 constructs were expressed in the transduced cells (Figure S7A), and immunofluorescence microscopy showed that the heterologously expressed Ttc26^wt^ and Ttc26^hop^ were imported into the cilium (Figure S7B). Results of the reporter assay demonstrated that heterologous expression of Ttc26^wt^ restored the SAG-dependent induction of the Gli reporter to nearly wild-type levels (Figure 4B), but that overexpression of Ttc26^hop^ had no significant effect (Figure 4B). These data indicate that the *Ttc26* mutation is the cause of the Hh signaling defect in the hop mouse line.

Activation of Gli3-F is followed rapidly by its proteasomal degradation [17], [18]. We therefore used Western blotting to examine whether the *Ttc26* mutation was associated with a defect in Gli3 processing. In *hop/hop* MEFs, the Gli3-F levels changed only minimally following SAG treatment, whereas in wild-type MEFs they declined significantly (Figure 4C and 4D). In addition, we found that the ratio of Gli3-F to Gli3-R was high in non-stimulated *hop/hop* MEFs (Figure 4C and 4E), suggesting that the *Ttc26* mutation also impaired the processing of Gli3-F into Gli3-R. These data are consistent with a cilium-dependent Hh signaling defect in *hop/hop* cells, because Gli3-F transport through the cilium is required for both the activation of Gli3-F and efficient production of Gli3-R [23], [67].

### The *hop* mutation does not affect accumulation of Gli at the ciliary tip but impairs its dissociation from Sufu

**Figure 5:**
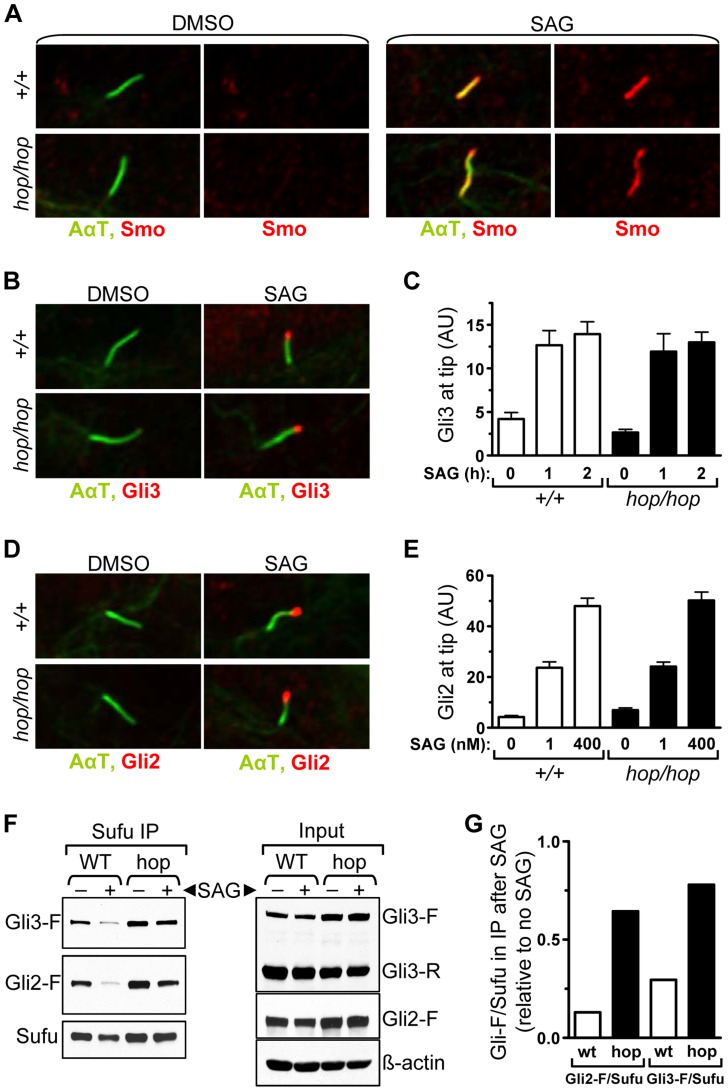
The *hop* mutation inhibits Gli-Sufu dissociation without altering Gli accumulation at the ciliary tip. (**A**) Immunofluorescence analysis of Smo (red) localization to primary cilium (AαT-labeled, green) in wild-type (+/+) and *hop/hop* MEFs following 4-h treatment with DMSO (0.02%) or SAG (400 nM) as indicated. (**B**) Immunofluorescence analysis of Gli3 (red) localization to primary cilium tip in wild-type (+/+) and *hop/hop* MEFs following 2-h treatment with DMSO (0.02%) or SAG (400 nM). (**C**) Quantitative analysis of Gli3 immunofluorescence signal intensities at the primary cilium tip in *+/+* and *hop/hop* MEFs following SAG treatment (400 nM) for the indicated times (mean ± SEM, n = 40–50). AU: arbitrary units. (**D**) Immunofluorescence analysis of Gli2 (red) localization to primary cilium tip in *+/+* and *hop/hop* MEFs following 2-day treatment with DMSO (0.02%) or SAG (400 nM). (**E**) Quantitative analysis of Gli2 immunofluorescence signal intensities at the primary cilium tip in *+/+* and *hop/hop* MEFs following 2-day treatment with the indicated concentrations of SAG (mean ± SEM, n = 45–55). AU: arbitrary units. (**F**) Gli-Sufu dissociation in *+/+* and *hop/hop* MEFs following 5-h incubation with SAG (400 nM) and the proteasome inhibitor bortezomib (2 µM) or with DMSO (0.02% negative control). Left panels show the relative amounts of Gli3-F, Gli2-F, and Sufu pulled down from the cell lysates using an anti-Sufu antibody. Right panels show the relative amounts of Gli3-F, Gli3-R, Gli2-F, Sufu and ß-actin (loading control) in the total cell lysates. (**G**) Ratios of band intensities of Gli-F and Sufu were calculated based on the immunoprecipitation results in the left column of panel F, and the Gli-F/Sufu ratios in the immunoprecipitated fractions of SAG-treated *+/+* (wt) and *hop/hop* (hop) cells are shown relative to the Gli-F/Sufu ratios in the immunoprecipitated fractions of non-treated cells.

We next probed the Hh pathway upstream of Gli3 processing in *hop/hop* cells by assessing the ciliary localization of Smo. Immunofluorescence experiments showed that SAG treatment led to an increase in the amount of Smo in the cilia of both *hop/hop* and wild-type MEFs (Figure 5A). Thus, the *Ttc26* mutation of hop mice did not block entry of Smo into the cilium. Next we used immunofluorescence to evaluate accumulation of the Gli protein at the ciliary tips of wild-type and *hop/hop* MEFs. Because Gli3 is degraded soon after stimulation with SAG (Figure 4C), its accumulation was measured after short treatments (i.e. 1 and 2 h, Figure 5B and 5C). In the case of the more stable Gli2 protein, accumulation was measured after both short (i.e. 2 h, Figure S8) and long (i.e. 48 h, Figure 5D and 5E) incubations with SAG. The results revealed that the *Ttc26* mutation did not block the SAG-induced transport of either Gli2 or Gli3 to the ciliary tip. To evaluate whether our immunofluorescence approach was suitable for detecting intermediate changes in the quantity of ciliary Gli2, we measured its accumulation at the ciliary tip after treating control and *hop/hop* MEFs with low and high concentrations of SAG. We found that high concentration of SAG (400 nM) led to a greater increase of the Gli2 signal at the tip than low concentration of SAG (1 nM), in both control and *hop/hop* MEFs (Figure 5E). Moreover, the Gli2 signal at the ciliary tip was stronger in *hop/hop* MEFs treated with 400 nM SAG than in wild-type MEFs treated with 1 nM SAG (Figure 5E), yet Gli reporter induction was milder in the former (Figure S9). Thus, the *Ttc26* mutation disrupts the correlation between the amount of Gli2 accumulated at the ciliary tip and the transcriptional output of the Hh pathway.

A subset of the cellular pool of Sufu protein that forms complexes with Gli is also transported to the ciliary tip following activation of the Hh pathway [7]. To evaluate whether the *hop* mutation affected Sufu accumulation in the cilium, we measured the intensity of Sufu immunofluorescence at the ciliary tip of *hop/hop* and control MEFs after treating them with SAG and DMSO (control) for 2 days (Figure S10). Our results showed that the SAG-induced accumulation of Sufu at the ciliary tip was not affected by the *hop* mutation, supporting the notion that the transport of Gli-Sufu complexes in the cilium was unimpaired.

Since the transport of Gli-Sufu complexes to the ciliary tip is followed by their dissociation [17], [18], we evaluated this signaling step in *hop/hop* and wild-type cells using an immunoprecipitation approach. MEFs were treated with SAG or DMSO (control) for 5 hours, at which point the Sufu-associated proteins were pulled down from the cell lysates using an anti-Sufu antibody. To prevent the degradation of Gli3 following its dissociation from Sufu, we treated the SAG-stimulated cells with the proteasome inhibitor bortezomib. Western blot analysis of the immunoprecipitated fractions revealed that, in the cases of both Gli3 and Gli2, dissociation from Sufu was reduced in the SAG-treated *hop/hop* vs. wild-type MEFs (Figure 5F and 5G). Thus, the Ttc26 deficiency in hop mice led to a general decrease in the dissociation of Gli proteins from Sufu.

## Discussion

In the present study, we show that the Hh signaling defect of hop mice is caused by a nonsense mutation in the *Ttc26* gene, which encodes a component of IFT complex B. Our analysis of the Hh pathway in *hop/hop* cells indicates that the signaling defect lies downstream of the accumulation of Gli at the ciliary tip, but upstream of its dissociation from Sufu.

How does the deficiency for Ttc26 function lead to impaired dissociation of the Gli-Sufu complex? Although the simplest interpretation of our data is that Ttc26 is an organizer of molecular interactions that lead to the dissociation of Gli from Sufu, this seems unlikely because neither our yeast two-hybrid screen nor previous high-throughput screens detected physical interactions between Ttc26 and Gli or Ttc26 and Sufu [68]–[70]. We therefore posit that Ttc26 facilitates Gli-Sufu dissociation indirectly, potentially by affecting the localization of other ciliary proteins. This hypothesis is supported, albeit indirectly, by the known role of Ttc26 in the motile cilium. Specifically, Ttc26 appears to be required for the correct localization of a group of proteins that form the inner dynein arm in the motile cilium; this is suggested by the fact that in hop mice approximately 40% of cilia in the trachea, ependyma, and oviduct lack inner dynein arms [43], and by the recent discovery of an association between the *Ttc26* mutation and a reduction in the levels of inner dynein-arm proteins in the flagella of the green alga *Chlamydomonas reinhardtii* [52]. Alternatively, it is possible that the *hop* mutation slows both the anterograde and retrograde transport of Gli proteins, because a balanced defect in the bidirectional transport would potentially temper the dissociation of Gli from Sufu without altering the amount of Gli at the ciliary tip. Although this alternative hypothesis cannot be ruled out, tracings of IFT particles in the flagella of the *Ttc26* mutant *C. reinhardtii* indicate that the velocity of IFT is not affected by the lack of Ttc26 [52]. Thus, the absence of Ttc26 is not likely to alter the structure of the primary cilium to the extent that the movement of motor proteins along the axoneme is impaired.

Some of the phenotypic features of hop mice have been described in various animal models of Gli protein deficiency. For example, *Gli1^−/−^; Gli2^+/−^* mice have a hopping gait [49], and *Gli3^+/−^* mice have preaxial polydactyly [48]. Thus, defective regulation of Gli proteins is the most likely cause of the hopping gait and polydactyly in the hop mouse. Nevertheless, the phenotype of hop mice is milder than that of *Gli2^−/−^* and *Gli3^−/−^* mice, which do not survive after birth [48], [49]. Our detection of residual induction of the Gli reporter gene in SAG-treated *hop/hop* MEFs (Figure 4A) is consistent with the relatively mild phenotype of hop mice. The residual Hh pathway activation in *hop/hop* cells suggests that Ttc26 is not absolutely necessary for Hh signaling. Alternatively, *hop* could be a hypomorphic mutation. The “modest” (5.7-fold) downregulation of the Ttc26 mRNA in *hop/hop* MEFs supports the notion that *hop* could be hypomorphic; however, the expression of the encoded Ttc26^hop^ protein was reduced much more dramatically (Figure 1D). This difference in the extent of downregulation of Ttc26^hop^ transcript and protein is compatible with NMD-dependent inhibition of Ttc26^hop^ expression. During NMD, the premature stop codon-containing mRNAs are not degraded immediately after the completion of pre-mRNA splicing; rather, their degradation occurs after a single round of pioneering translation. The temporal lag between splicing and degradation leads to the steady-state abundance of aberrant mRNAs at levels which are “only” 3- to 10-fold below the expression of their wild-type counterparts [55], [71]. Our inability to detect truncated Ttc26 protein in *hop/hop* cells could reflect the very low protein output of the pioneering translations of Ttc26^hop^ mRNA molecules.

Among the various mouse models that involve deficiencies in complex B subunits, the orpk and gt mice exhibit the most hop-like phenotypes. These mice carry hypomorphic alleles of two complex B protein-encoding genes, *Ift88* and *Ift80*, respectively. Homozygosity for the *Ift88^Orpk^* and *Ift80^gt^* alleles leads to postnatal growth retardation, preaxial polydactyly and incomplete pre-weaning lethality [37], [72], [73]. In the hop and *Ift80^gt/gt^* mice, the Hh signaling defects are not accompanied by pathological changes in the kidney. In the *Ift88^Orpk/Orpk^* mice, by contrast, the Hh signaling defect is accompanied by polycystic kidney disease. Cyst formation correlates with deformities in the cilia of *Ift88^Orpk/Orpk^* and *Ift80^gt/gt^* mice, with the *Ift88^Orpk/Orpk^* genotype – but not the *Ift80^gt/gt^* genotype – being associated with the shortening of primary cilia. Thus, the absence of polycystic kidney disease in the hop mouse is consistent with the lack of cilium shortening in *hop/hop* MEFs. Although ciliary trafficking of Gli has not been examined in the *Ift88^Orpk/Orpk^* and *Ift80^gt/gt^* cells, the ciliary localization of Smo has been found to be dysregulated in *Ift88^Orpk/Orpk^* MEFs [74]. Thus, the *orpk* mutation appears to affect the Hh pathway further upstream than the *hop* mutation.

The *Ttc26* mutation led to an elevated hearing threshold in the hop mice; however, unlike the absence of many other ciliary proteins, the Ttc26 defect did not disrupt the planar polarity of outer hair cells (Figure S3). Furthermore, histological analysis of the middle ear of *hop/hop* mice did not reveal signs of inflammation (Figure S11), a frequent consequence of defects in the motile cilia. Because a complete lack of Hh signaling has been shown to cause agenesis of the middle ear ossicles and the cochlear duct [75], [76], we speculate that reduced Hh signaling in hop mice could potentially impair hearing through as yet undetected alterations in the structures of the middle ear ossicles or the expression of cochlear genes.

The biological relevance of Ttc26 was recently evaluated in zebrafish [52], [77], and comparison of the zebrafish and mouse models of Tc26 deficiency reveals both similarities and differences in Ttc26 function. In both species the motile cilia are defective, as indicated by reduced ciliary beating in Ttc26 knockdown zebrafish [52], [77] and the partial loss of inner dynein arms in the hop mouse [43]. However, abnormal patterning has not been reported in the Ttc26 knockdown zebrafish, and thus Ttc26 may not be necessary for Hh signaling in fish. This notion is supported by the recent finding that the morpholino-dependent knockdown of various subunits of the IFT complexes in zebrafish leads to minimal Hh signaling defects but severely deformed cilia [78], [79]. Another difference between the two animal models is the impact of the Ttc26 deficiency on the length of cilia. In the Ttc26 knock-down zebrafish the motile cilia are shorter than normal [52], [77], whereas in the hop mouse the lengths of neither the motile [43] nor the primary cilia (Figure 3) are generally decreased – the only exception being the sperm flagellum [42]. This discrepancy in cilium length cannot be explained simply by species differences in ciliogenesis, because the shRNA-mediated knockdown of Ttc26 in mouse cell lines also leads to the formation of short cilia [77], [80]. The unique feature of the hop cells is the presence of a premature stop codon towards the end of the coding region in the Ttc26 mRNA. Therefore, we suggest that very low expression of the truncated Ttc26 protein could be responsible for the lack of cilium shortening in the hop mouse.

Models describing IFT function in Hh signaling have developed rapidly over the past 10 years. After key Hh signaling molecules were detected in the primary cilium, IFT defects were proposed to affect the Hh pathway by causing structural changes in the axoneme, a model supported by the finding that a combination of anterograde and retrograde trafficking defects results in milder structural and signaling anomalies in the cilium than does either in isolation [40]. This model was further developed with the discovery that, in mice deficient for the IFT complex B subunit Ift25, the ciliary structure is intact but ciliary trafficking of Ptch1 and Smo is dysregulated [34]. This indicated that the contribution of complex B to Hh signaling is not limited to maintenance of the ciliary microtubule track. The detailed analysis of a series of mouse lines carrying various hypomorphic and null alleles of the complex A subunit Ift144 has also suggested that changes in the ciliary structure alone do not fully explain the Hh signaling defects of the mutant mice [31]. The results we present here show that the Ttc26 component of IFT complex B is necessary for efficient coupling between the ciliary accumulation of Gli and its activation. We thus propose that the Ttc26 defect of hop mice reveals a novel role for IFT complex B in Hh signaling, downstream of the maintenance of ciliary structure and the facilitation of Smo trafficking.

## Materials and Methods

### Ethics statement

Mice were euthanized according to the current AVMA guidelines. Experimental procedures were approved by the Animal Care and Use Committee of the University of Iowa (protocol#: ACURF 1303050).

### Genetic, histological, and ABR analysis of hop mice

Hop mice (BALB/cByJ genetic background) were obtained from The Jackson Laboratory. The *hop* allele was mapped by sequencing 15 PCR-amplified genomic regions that contain BALB/c specific SNPs (SNP accession numbers and PCR primers are listed in Table S1). The transcripts of candidate genes were RT-PCR amplified from testis RNA, using the PCR primers listed in Table S2, and sequenced without subcloning. Our routine genotyping procedure detected the single nucleotide difference between the *hop* and *WT* alleles based on the elimination of a *Bpu10I* restriction site from *Ttc26* by the *hop* mutation. In brief, a 618-bp long genomic segment containing the *hop* mutation site was PCR amplified using the PCR primers 5′-dTACTGCTTTTGAGGAGACTAGGG-3′ and 5′-dGGATGATGGAACTAGTCACGGG-3′, the reactions were digested with *Bpu10I* (New England BioLabs), and the digestion products were resolved on 1% agarose gels (Figure S12). Kidneys and eyes from ∼1 year old mice were fixed, paraffin-embedded, sectioned, and stained with hematoxylin and eosin. Fore and hind limbs from newborn mice were stained with Alcian Blue and Alizarin Red S as previously described [81]. Hematoxylin and eosin stained cochlear sections and phalloidin-Alexa Fluor 488 stained organ of Corti samples were prepared as previously described [82]. The ABR thresholds of mice were measured at postnatal day 21–28, using a previously described open-field system and broadband click stimuli [83].

### Constructs, viral vectors, and HEK293 cell culture

The Gli^x^8-luciferase reporter cassette was kindly provided by Dr. Michael K. Cooper (Vanderbilt University Medical Center, Nashville, TN); all other constructs were generated from mouse RNA. The Pfu DNA polymerase was used under standard reaction conditions, with primers listed in Table S3, to amplify the entire coding regions of the Ift46, Ttc26^wt^, and Ttc26^hop^ transcripts, the 5′ coding region of the Shh mRNA (1–594 nucleotides), and the entire Gli^x^8-luciferase reporter cassette. The amplified DNA fragments were subcloned into yeast expression vectors (pGBKT7 and pGADT7), the mammalian expression vector pcDNA3.1, and two adenoviral shuttle vectors (pacAd5-CMV and the promoterless pacAd5). Adenoviral particles were generated by the Gene Transfer Vector Core of the University of Iowa and by ViraQuest Inc. (North Liberty, IA). The Gli^x^8-luciferase encoding adenovirus contained a PGK-EGFP expression cassette; all other viral vectors lacked GFP. HEK293 cultures were maintained and transfected as previously described [84]. Shh-conditioned medium was harvested from HEK293 cells 3 days after transfecting them with the Shh-encoding plasmid. The harvested medium contained 2% FBS (Hyclone), and it was used in MEF cultures after 5-fold dilution with DMEM.

### Yeast two-hybrid screens

The Matchmaker Gold Yeast Two-Hybrid system was used, according to the manufacturer's instructions (Clontech), to screen human testis, mouse embryo, and universal mouse cDNA libraries (Clontech) for genes whose products interact with Ttc26^wt^. Verification of the Ift46-Ttc26^wt^ interaction was carried out in Y2HGold yeast (Clontech) co-transformed with plasmids encoding Ift46 (in pGADT7 vector) and either the WT or mutant form of Ttc26 (in pGBKT7), using the Yeastmaker Yeast Transformation System (Clontech). Following transformation, the yeast was spread on X-α-Gal-supplemented double dropout medium lacking leucine and tryptophan.

### Immunofluorescence on mouse embryo sections

Pregnant mice were euthanized at E10.5. Embryos were removed from the amniotic cavity, fixed in 4% PFA for 4 h at 4°C, cryoprotected in 30% sucrose solution, and embedded in Optimal Cutting Temperature compound. Transverse cryosections through the lumbar region were re-fixed with pre-chilled 4% PFA for 10 min at RT, permeabilized with 0.1% Triton X-100 for 10 min, blocked with 5% normal goat serum (Sigma) and incubated with antibodies against FoxA2 (Abcam; 1∶250 dilution), HB9 (Developmental Studies Hybridoma Bank, University of IA; 1∶30 dilution), Nkx2.2 (Developmental Studies Hybridoma Bank, University of IA; 1∶10), acetylated α-tubulin (1∶1000, Sigma), or Arl13b (1∶500, Protein Tech). Secondary antibodies were labeled with Alexa fluor 488 and Alexa fluor 568 (Invitrogen, 1∶500), and fluorescence was visualized using a confocal microscope (LSM-510, Carl Zeiss Inc.).

### MEF culture, immunostaining of cilia, and cilium length measurement

MEFs were isolated from E10.5 embryos as described previously [85]. In brief, decapitated and eviscerated embryos were pressed through 18-gauge needles twice, and pipetted onto 0.2% gelatin-coated plates in high-glucose DMEM containing 10% FBS, penicillin (100 units/ml), streptomycin (100 µg/ml), and 4 mM L-glutamine. MEF cultures were maintained for 4–6 passages. For immunofluorescence experiments, MEFs were seeded onto glass coverslips and cultured to near confluency. Cilium formation was induced by serum starvation (0.4% FBS in DMEM) for 48 h. When SAG treatment was used, cells were serum starved for 16–24 h prior to addition of the SAG-containing medium. Cultures were fixed in 4% PFA for 15 min, permeabilized with 0.2% Triton X-100 for 7 min, and blocked in 5% normal donkey serum (Sigma), 5% normal goat serum (Sigma), or 1% BSA, depending on the primary antibody used (see Table S4). The sources, catalogue numbers, and dilutions of the primary antibodies against acetylated α-tubulin, Arl13b, Ttc26, Ift46, Smo, Gli2, Gli3, and Sufu are also listed in Table S4. The secondary anti-mouse, anti-rabbit, and anti-goat antibodies (1∶500, Invitrogen) were labeled with Alexa fluor 488 or Alexa fluor 568. For analysis of the percentage of ciliated MEFs, cells were incubated with the nuclear stain ToPro-3 (1∶2000; Invitrogen) for 10 min immediately before the slides were mounted. Images were obtained using a confocal microscope (LCM-510, Carl Zeiss Inc.). Intensity of the Gli signal at the ciliary tip (400-pixel area) was measured using the ZEN software (Carl Zeiss Inc.). Cilium length was measured in 4% PFA-fixed and acetylated α-tubulin-stained samples at 63× magnification (0.03 µm×0.03 µm pixel size), by line segment tracing and spline fitting in Image J.

### Adenoviral transduction of MEFs

MEFs were seeded onto glass coverslips and plastic dishes in DMEM containing 10% FBS. Approximately 16 h after seeding, the indicated adenoviral particles were added to MEFs at 40 multiplicity of infection (MOI), in DMEM containing 1.8% FBS and polybrene (2.75 µg/ml). Cells were incubated with adenoviruses for 4 h and then allowed to recover for 4 h in 10% FBS-containing DMEM. The Hh pathway was activated by incubating MEFs with SAG (1–400 nM) in 0.4% FBS-containing DMEM for 48 h. Control cultures were incubated with DMSO (0.02%) instead of SAG. The transduced MEFs were used for immunostaining, protein extraction, and luciferase assay.

### Luciferase assay

Control and adenovirus transduced MEFs were incubated with SAG (1–400 nM) or DMSO (0.02%) in 0.4% FBS-containing DMEM for 48 h before lysis with Reporter Lysis Buffer (Promega) according to the manufacturer's protocol. Cell lysates were cleared by brief centrifugation, and luciferase activity in the supernatants was measured using the Luciferase Assay System (Promega) following the manufacturer's instructions. Luminescence was quantified with a Victor3 1420 Multilabel Plate Counter (Perkin Elmer). To facilitate the evaluation of relative transduction efficiency, the Gli reporter-encoding adenovirus was engineered to contain a PGK-EGFP expression cassette. GFP fluorescence in the supernatants of cell lysates was measured with the Victor3 1420 Multilabel Plate Counter and was used for normalization of luciferase activity.

### Co-immunoprecipitation and Western blotting

MEFs were serum starved for 24 hours before treatment with 400 nM SAG or 0.02% DMSO (control) for 0–5 h in DMEM plus 0.4% FBS. Cells were lysed in a previously described buffer [18] containing 50 mM Tris (pH 7.5), 300 mM NaCl, 2% NP-40, 0.25% deoxycholate, 10 mM N-ethylmaleimide, 1 mM DTT, 10 µL/mL P8340 (Sigma), 1 mM PMSF, 10 µg/mL chymostatin, and PhosSTOP Phosphatase Inhibitor Cocktail (Roche). When bortezomib (2 µM) was added to the cell culture medium, its concentration was maintained in the lysis buffer. The cell lysates were passed through insulin needles ∼5 times and cleared by centrifugation. Aliquots from the supernatant were used directly for Western blotting, or were used for immunoprecipitation. In the latter case, the samples were incubated with 7 µg of an anti-Sufu antibody (Santa Cruz, sc-28847) overnight at 4°C, and immunocomplexes were pulled down using the Dynabeads Protein G Immunoprecipitation kit (Invitrogen). The pulled-down and input fractions were immunoblotted using the following antibodies: anti-Gli3 (R&D, AF3690), anti-Gli2 [86] (kindly provided by Dr. Jonathan T. Eggenschwiler, University of Georgia, Athens, GA), anti-Sufu (Santa Cruz, sc-10933), and anti-ß-actin (Santa Cruz, sc-1616).

For the FL-Ttc26 and HA-Ift46 co-immunoprecipitation experiments, lysis buffer (Buffer A) consisted of PBS (pH 7.2), 5 mM EDTA, 0.5% Triton X-100, 10 µL/mL P8340, 1 mM PMSF, and 10 µg/mL chymostatin. Transfected HEK293 cells were scraped into ice-cold Buffer A and sonicated (40% amplitude, 2×6 s at 4°C). The lysates were cleared by centrifugation, and aliquots of the supernatant were used directly as “input control” during Western blotting, or were used for immunoprecipitation. In the latter case, the samples were incubated with 7 µg of a monoclonal anti-flag antibody (Sigma, M2 clone) and immunocomplexes were pulled down using protein G agarose beads as previously described [84]. The pulled-down and input fractions were immunoblotted using the following antibodies: anti-HA (Abcam, ab9134) and anti-flag (Abcam, ab1162). Airway epithelial cells were harvested from 5–6 mouse tracheas by pronase digestion, as described previously [87]. Protein was extracted from airway epithelial cells, testes, Ttc26-transfected HEK293 cells, and Ttc26-transduced MEFs using Buffer A. Protein extracts were immunoblotted with anti-Ttc26 (Novus Biologicals, NBP1-84034) and anti-ß-actin (Santa Cruz, sc-1616) antibodies.

### Real-time PCR

Total RNA from serum-starved MEFs was isolated using the TRIzol reagent (Invitrogen), and reverse transcribed using SuperScriptIII (Invitrogen). Real-time PCR was performed using PerfeCTa SYBR Green Fastmix (VWR), a Mastercycler ep realplex PCR machine (Eppendorf), and the following primers: Ttc26 forward 5′-dTGGCCAGGAAATGGGTTCAAGG-3′ and reverse 5′-dACTAGCTGATCCTCCTACCAACTG -3′, Gapdh forward 5′-dCGTCCCGTAGACAAAATGGT-3′ and reverse 5′-dGAATTTGCCGTGAGTGGAGT-3′. The relative mRNA expression values were determined using the ΔΔC_T_ method [88].

## Supporting Information

Figure S1 Real-time RT-PCR quantification of Ttc26 mRNA expression in wild-type and *hop/hop* MEFs. The level of the Ttc26 mRNA was normalized to that of the control mRNA (Gapdh) in each sample. The average expression level of Ttc26 in *hop/hop* MEFs is shown relative to that of wild-type (*+/+*) cells. Results represent mean ± SEM (n = 3, unpaired *t*-test with Welch's correction, **p = 0.0043).
(EPS)

Figure S2 Histological analysis of the inner ears of *+/+* and *hop/hop* mice. (**A**, **B**) Hematoxylin- and eosin-stained cross sections of cochleas from *+/+* (**A**) and *hop/hop* (**B**) mice at postnatal day 30. Lower panels show the areas boxed in the upper panels at higher resolution. Scale bars are 250 µm in the upper panels and 100 µm in the lower panels.
(EPS)

Figure S3 Lack of planar polarity defects in the organ of Corti of *hop/hop* mice. Organ of Corti whole-mount preparations from *+/+* (upper panel) and *hop/hop* (lower panel) mice were stained with phalloidin-Alexa Fluor 488 at postnatal day 30. The phalloidin staining visualizes mainly the stereociliary bundles of outer hair cells (arrowheads) and inner hair cells (arrows), the cell-cell junctions, and the cytoskeleton of pillar cells (block arrows). Scar formation at the sites of missing hair cells is indicated by asterisks; sporadic loss of outer hair cells at 1 month of age is typical of the BALB/c mouse strain. Scale bars: 20 µm.
(EPS)

Figure S4 Histological analysis of kidney and retinal sections from 1-year old *hop/+* and *hop/hop* mice. (**A**) Representative kidney sections, revealing that both heterozygous and homozygous mutants lack cysts. Scale bars: 200 µm. (**B**) Representative retinal sections, revealing slightly thinner retina and outer segment layer in the eyes of hop mice, but no significant photoreceptor degeneration which would be reflected by a loss of nuclei in the outer nuclear layer. The following layers of the retina are indicated: ganglion cell layer (GCL), inner nuclear layer (INL), outer nuclear layer (ONL), inner segment (IS), outer segment (OS), retinal pigment epithelium (RPE), and choroid (CH). Scale bars: 50 µm.
(EPS)

Figure S5 The C-terminal truncation of the Ttc26 protein impairs its interaction with Ift46. Statistical analysis of Ift46 band intensity ratios in the Western blotted fractions shown in Figure 3B (i.e. immunoprecipitated and input). The indicated combinations of FL-Ttc26^wt^, FL-Ttc26^hop^, and HA-Ift46 were transfected into HEK293 cells, and the immunoprecipitated fractions were generated using an anti-FL antibody. HA-Ift46 was Western blotted using an anti-HA antibody. Only ∼10% of the cell lysates was loaded and quantified as input. Results represent mean ± SEM (n = 3, unpaired *t*-test, **P = 0.007).
(EPS)

Figure S6 The *hop* mutation does not lead to shortened cilia in the embryonic mesenchyme. Statistical analysis of cilium length in the mesenchyme of *hop/+* and *hop/hop* mouse embryos (E10.5). Results represent mean ± SEM (n = 60 cilia per genotype, unpaired *t*-test with Welch correction, ***P<0.0001).
(EPS)

Figure S7 Evaluation of heterologous Ttc26 expression in *hop/hop* cells. (**A**) Immunoblot analysis of Ttc26 expression in *hop/hop* MEFs transduced with adenoviral vectors encoding Ttc26^wt^ or Ttc26^hop^ as indicated. Cells not transduced with a Ttc26-encoding adenovirus (no Ttc26) served as negative controls. (**B**) Immunofluorescence analysis of Ttc26 expression (red) in the cilia of *hop/hop* MEFs transduced with the indicated adenoviral vectors. Negative control *hop/hop* MEFs were not transduced with adenoviral vectors (no Ad). The ciliary axonemes were visualized by immunolabeling of acetylated-α-tubulin (AαT, green). The anti-Ttc26 antibody used for the immunostaining experiments recognizes a region of Ttc26 that is present in Ttc26^hop^.
(EPS)

Figure S8 Gli2 accumulation at the cilium tip in *+/+* and *hop/hop* MEFs after short SAG treatment. Quantitative analysis of Gli2 immunofluorescence signal intensities at the primary cilium tip in *+/+* and *hop/hop* MEFs following 2-h treatment with 0.02% DMSO (-, negative control) or 400 nM SAG (mean ± SEM, n = 40). AU: arbitrary units. Kruskal-Wallis test, *P*<0.0001; *post hoc* Dunn's test, **P<0.01, ***P<0.001. The ciliary Gli2 immunofluorescence did not differ significantly between *+/+* and *hop/hop* cells treated in similar fashion.
(EPS)

Figure S9 SAG-dependent induction of the Gli^x^8-luciferase reporter gene in *+/+* and *hop/hop* MEFs. Induction of an adenovirus-delivered Gli^x^8-luciferase reporter gene in *+/+* (open squares) and *hop/hop* (closed circles) MEFs following 2-day incubation with SAG at the indicated concentrations (mean ± SEM, n = 3–7). The increase in luciferase expression is shown relative to that in DMSO controls (0 nM SAG). Constitutive GFP expression from the Gli^x^8-luciferase-encoding viral vector was used for normalization.
(EPS)

Figure S10 The *hop* mutation does not prevent Sufu accumulation at the tip of the primary cilium. (**A**) Immunofluorescence analysis of Sufu (red) localization to the primary cilium tip in *+/+* and *hop/hop* MEFs following 48-h treatment with 0.02% DMSO (negative control) or 400 nM SAG. The ciliary axonemes were visualized by immunolabeling of acetylated-α-tubulin (AαT, green). (**B**) Quantitative analysis of Sufu signal intensities at the primary cilium tip in the experiments described in panel A (mean ± SEM, n = 45–50). AU: arbitrary units. One-way analysis of variance (ANOVA), *P*<0.0001; *post hoc* Bonferroni's test, ***P<0.001. The ciliary Sufu immunofluorescence did not differ significantly between *+/+* and *hop/hop* cells treated in similar fashion.
(EPS)

Figure S11 Lack of middle ear inflammation in *hop/hop* mice. Hematoxylin- and eosin-stained cross sections of decalcified temporal bones from a *+/+* mouse (upper panel) and a hearing impaired *hop/hop* mouse (lower panel) at postnatal day 60. Scale bars: 500 µm.
(EPS)

Figure S12 Genotyping for the *hop* mutation. The *hop* and wild-type alleles were distinguished by PCR amplification and subsequent *Bpu10I* digestion of a fragment of the *Ttc26* gene. Lanes show the genotyping results obtained using DNA samples from *+/+*, *hop/+*, and *hop/hop* mice. Arrows indicate the calculated fragment sizes in base pairs (bp).
(EPS)

Table S1 Primers used for the amplification of genomic segments containing selected BALB/c-specific SNPs from chromosome 6.
(XLSX)

Table S2 Primers used for the amplification of transcripts encoded by candidate genes at the *hop* locus.
(XLSX)

Table S3 Primers used for the production of gene expression constructs.
(XLSX)

Table S4 Antibodies and immunostaining conditions used for the visualization of ciliary and signaling proteins.
(XLSX)
